# *Monomorium sahlbergi* Emery, 1898 (Formicidae, Hymenoptera): a cryptic globally introduced species

**DOI:** 10.3897/zookeys.979.55342

**Published:** 2020-10-27

**Authors:** Peter Boer, Ana Carolina Loss, Frederique Bakker, Kevin Beentjes, Brian L. Fisher

**Affiliations:** 1 Department of Terrestrial Zoology, Naturalis Biodiversity Center, PO Box 9517, 2300 RA Leiden, The Netherlands Naturalis Biodiversity Center Leiden Netherlands; 2 Entomology, California Academy of Sciences, 55 Music Concourse Drive, San Francisco, CA 94118, USA California Academy of Sciences San Francisco United States of America; 3 National Institute of the Atlantic Forest (INMA), Santa Teresa, ES, Brazil National Institute of the Atlantic Forest Santa Teresa Brazil

**Keywords:** CO1, invasive species, *Monomorium dichroum*, *Monomorium pharaonis*

## Abstract

The discovery in the Netherlands in a shipping container of the ant *Monomorium sahlbergi* Emery, 1898, a species similar to the invasive pharaoh ant *M. pharaonis* (Linnaeus, 1758), led to a quest to better define the distribution of this species, which was initially obscure due to uncertain specimen identifications. Here it is shown that *M. sahlbergi*, like *M. pharaonis*, is found worldwide, almost certainly as a result of introductions. Including quarantine interceptions, this species is recorded from seven global biogeographic regions, but its established outdoor distribution is currently limited to the tropics and subtropics. *Monomorium dichroum* Forel, 1902 is here presented as a junior synonym of *M. sahlbergi***syn. nov.** based on morphometric and CO1 analyses.

## Introduction

Broadening transport networks and rising demand for commodities have led to increases in alien species worldwide (Hulme 2009), including ants (Suarez et al. 2001; Bertelsmeier et al. 2017). In the Netherlands, for example, a relatively large number of non-native ant species are being recorded owing in part to the shipments of plant material imported into the country (Boer and Vierbergen 2008).

A concerted effort is underway to identify ant species introduced into the Netherlands, whether they are established or found during import inspections. Thus far 120 species have been identified (Boer et al. 2018). Many of these introduced species are poor colonisers and have not been able to establish and/or spread after arriving (Boer and Vierbergen 2008). The actual number of introduced species is almost certainly greater; some specimens are impossible to identify due to a lack of suitable identification keys and uncertainty about the origin of the ants. Limited identification tools and training increase the chances that species names are ascribed incorrectly, especially in the case of closely related species. In this work we describe an example of one invasive species remaining hidden in the guise of another, more common species. The case concerns two closely related species of the genus *Monomorium*, of which one, *M. pharaonis* (Linnaeus, 1758), is considered the most notorious pest ant species in the world (Wetterer 2010). In the Netherlands, *M. pharaonis* is the first recorded tramp ant species; the oldest specimen is dated 1877 (Boer and Vierbergen 2008).

On 2 June 2014, the pest controller A.J.A. Heetman intercepted ants found in a shipping container at a distribution company in the Netherlands and sent them to the first author. The shipping container, filled with glycine for the food industry, came from a chemical plant in Wuyi, Hengshui, Hebei, China. The intercepted ants appeared similar to the well-known and globally common tramp species *M. pharaonis*, but differed in their black gaster. While trying to identify the specimens, we came across images of identical specimens on AntWeb (http://www.antweb.org), where they were recorded under the provisional name *M. pharaonis*_nr (CASENT0173275, CASENT0246074) and *M. bicolor* complex (CASENT0178876).

**Figure 1.**
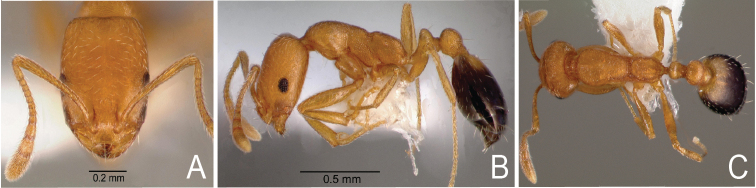
*Monomorium sahlbergi* from Sacramento, USA, imported from Thailand. Worker, CASENT0005783**A** frontal view **B** lateral view **C** dorsal habitus.

**Figure 2.**
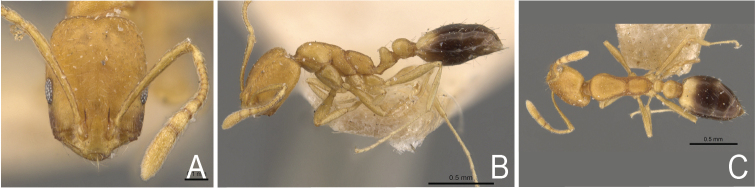
*Monomorium dichroum*, syntype from Mumbai, India. Worker, CASENT0908718**A** frontal view **B** lateral view **C** dorsal habitus.

**Figure 3.**
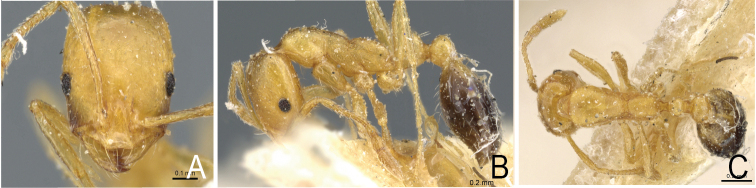
*Monomorium sahlbergi*, syntype from Jericho, Palestine. Worker, CASENT0904576**A** frontal view **B** lateral view **C** dorsal habitus.

Further comparison of our specimens with the images from AntWeb convinced us that the ants discovered in the Hebei shipping container were a previously described species, *M. dichroum* Forel, 1902 (Figs 1–3). *Monomorium dichroum* was reported as only known from India (type locality) (Imai et al. 1984, Bharti 2015) and China (Guénard and Dunn 2012).

Further exploration of similar species on AntWeb, however, suggested our specimens, and *M. dichroum* for that matter, were identical to *M. sahlbergi* Emery, 1898, a little-known species described from Israel. We set out to ascertain the true identity of our specimens and determine whether *dichroum* and *sahlbergi* are two distinct species.

## Materials and methods

Available descriptions of all *Monomorium* species occurring in the area between Saudi Arabia in the west and China in the east were consulted. Syntype material of *M. dichroum* and *M. sahlbergi* were requested and investigated. *Monomorium pharaonis*, M. cf. pharaonis, M. nr. pharaonis, and *M. bicolor*-complex ants identified from the collection of CASC and RMNH were investigated. In total, we examined hundreds of specimens from the Netherlands, France, Germany, Israel, Saudi Arabia, United Arab Emirates, Oman, Yemen, Seychelles, Papua, Nepal, New Zealand, Western Australia, Myanmar, Taiwan, China, Ivory Coast, Cameroon, Madagascar, Indonesia, Panama, Mexico, Trinidad, Netherlands Antilles, and the United States of America.

For morphometrical comparisons, 16 workers of *M. pharaonis* were examined (all in the collection of Naturalis Biodiversity Center, RMNH). The size and shape characters of these workers were quantified (Table 1) and reported as lengths or indices. All measurements are in millimetres. The numeric characters and abbreviations are defined below.

**CI** Cephalic Index (CW/CL) ×100.

**CL** Maximum cephalic length in median line.

**CW** Maximum cephalic width, across eyes.

**EYI** Eye Index (maximum eye length / CW) ×100.

**Omm** Number of ommatidia across the widest diameter of the eye.

**PI** Petiole Index (Maximum width of petiole / maximum width postpetiole) ×100.

**PrI** Promesonotal Index (Promesonotal width / CW) ×100.

**SI** Scape Index (Maximum straight line scape length excluding articular condyle / CW) ×100.

The examined specimens in this study are deposited in the following institutions:

**CASC** California Academy of Sciences, USA

**MHNG** Museum d’Histoire Naturelle, Geneva, Switzerland

**MSNG** Museo Civico di Storia Naturale ‘Giacomo Doria’, Genova, Italy

**RMNH** Naturalis Biodiversity Center, Leiden, the Netherlands (the former Rijksmuseum van Natuurlijke Historie)

**TAMU** Texas A & M University, Texas, USA

**UCDC** R.M. Bohart Museum of Entomology, University of California, Davis, USA

**NZAC** New Zealand Arthropod Collection, D.S.I.R., Auckland, New Zealand

### DNA sampling

We sequenced 654 base pairs (bp) of mitochondrial cytochrome oxidase I (COI) gene from 39 *Monomorium* specimens previously identified as *M. pharaonis*, *M. dichroum*, or *M. sahlbergi*. DNA extraction and COI sequencing were performed at University of Guelph (Ontario, Canada) and Naturalis Biodiversity Center (Leiden, the Netherlands), following the protocol described in Fisher and Smith (2008). All sequences are available at GenBank and Appendix 1. Phylogenetic analyses also included 20 *Monomorium* sequences from GenBank and two sequences as outgroup (*Huberia striata* and *Podomyrma* sp.), see Appendix 1 for sequence details.

*Molecular phylogenetic inference.* Sequences were aligned using Geneious 11.1.5 (Biomatters Ltd.). The phylogenetic tree was inferred in MEGA7 using maximum likelihood and 100 bootstrap replicates. Nucleotide substitution model selection and genetic p-distance calculation were also performed using MEGA7 (Kumar and Tamura, 2016). The best fit model selected under the corrected Akaike Information Criteria (AICc) was GTR+G+I.

## Results

### CO1

The phylogenetic tree recovered sequences of *M. dichroum* and *M. sahlbergi* in the same clade (Fig. 5), showing low within-clade genetic distance (1.0%). Genetic distance among sequences previously identified as *M. dichroum* and *M. sahlbergi* was also low (1.3%). All *M. pharaonis* sequences clustered together, showing 0.3% within genetic distance and 16.5% genetic distance between this and the *M. dichroum* + *M. sahlbergi* clade.

### Morphological comparisons

*Monomorium dichroum* and *M. sahlbergi* show similar colouration, especially with regard to the infuscate genae and the light spot on the posterior side of the gaster. Morphometrically, these ants are identical. None of the regression analyses of various morphometrical data, such as cephalic width versus cephalic length, scape length, maximum width of postpetiole, width of postpetiole versus width of petioles, comparisons between the cephalic index versus eye index, versus petiole index, versus scape index, and versus promesonotal index, showed any difference. The number of ommatidia across the widest diameter of the eye was the same. Nor could we find any differences in pilosity and pubescence. The surface sculpturing of the head, mesosoma, nodes, and gaster were the same.

**Figure 4.**
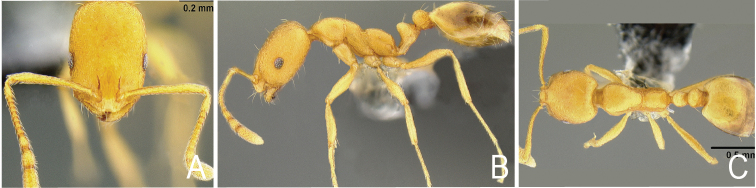
*Monomorium pharaonis* from Nampar Macing, Indonesia. Worker, CASENT0171086**A** frontal view **B** lateral view **C** dorsal habitus.

Both *Monomorium sahlbergi* and *M. pharaonis* belong to the *salomonis* group, as defined by Bolton (1987). For a detailed description of *M. pharaonis* see Heterick (2006). Morphometrically *M. sahlbergi* is similar to *M. pharaonis* (Table 1). Compared to the workers of *M. pharaonis*, 1/4 instead of 2/3 of the first gastral tergite (abdominal segment 4) is light-coloured; the structure of the frontal side of the head is strigulate rather than reticulate; the mesonotal groove is shallower; the pronotum and metanotum are higher than the propodeum in *M. pharaonis* as opposed to equally high in *M. sahlbergi* and promesonotal setae are missing on the mesosoma, in *M. pharaonis* two to six (Figs 1–4). Note that in *Monomorium* specimens the setae are quite stiff and break easily, thus reducing utility of this character in some specimens.

**Table 1.** Morphometric data of workers of *Monomorium dichroum*, *M. sahlbergi*, and *M. pharaonis*. Arithmetic mean in parentheses.

	***M. dichroum* (n = 48)**	***M. sahlbergi* (n = 32)**	***M. pharaonis* (n = 16)**	***M. pharaonis* (n = 50) from Bolton 1987**
CW	0.41–0.54 (0.44)	0.40–0.44 (0.42)	0.41–0.48 (0.44)	0.40–0.48
CL	0.49–0.66 (0.54)	0.49–0.54 (0.51)	0.52–0.59 (0.56)	0.52–0.60
CI	79–85 (82)	79–85 (81)	75–84 (80)	73–80
EYI	19–24 (21)	19–26 (22)	18–20 (19)	18–21
Omm	7–10 (9)	7–11 (9)	7–9 (8)	5–7
PI	67–86 (74)	64–73 (69)	71–82 (78)	–
SI	102–110 (103)	103–110 (106)	105–117 (109)	105–117

**Figure 5.**
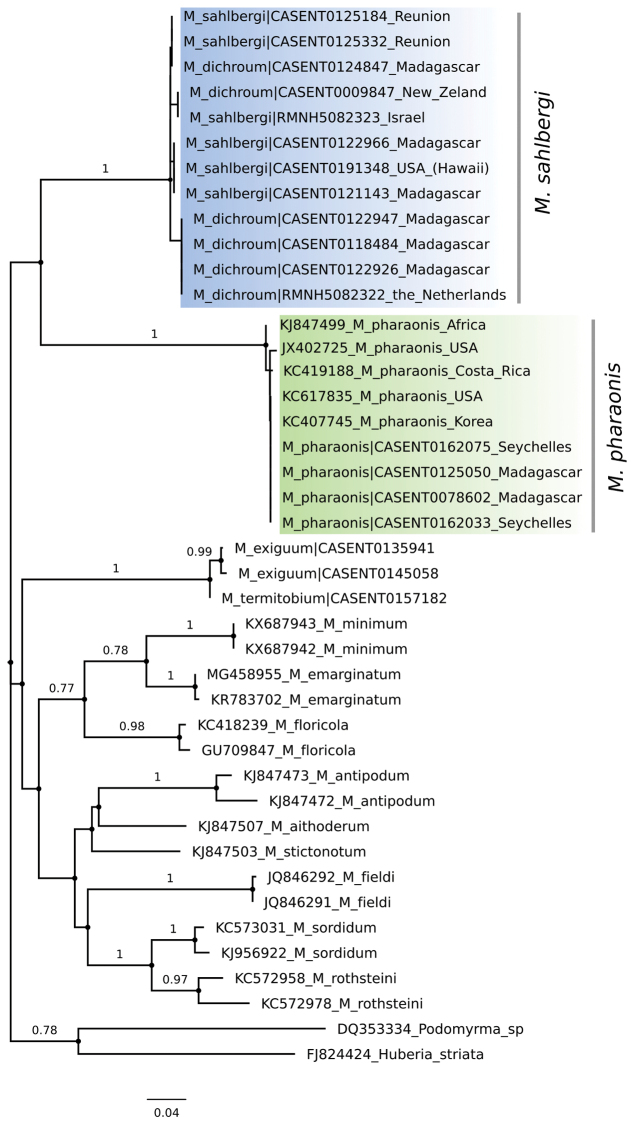
Maximum likelihood phylogeny of *Monomorium*COI sequences. Blue clade corresponds to *M. sahlbergi* and green clade to *M. pharaonis*. Values associated to nodes correspond to bootstrap values.

### Taxonomic implications

#### Monomorium sahlbergi

Taxon classificationAnimaliaHymenopteraFormicidae

Emery

05B31068-1633-56A2-AE1B-D215CD786FF6

Monomorium sahlbergi Emery, 1898: 131. Syntype worker, ergatoid queen: [Jerico] Jericho, Palestine (J. Sahlberg) (MSNG; worker, unique specimen code CASENT0904576; ergatoid queen, CASENT0904577) [examined].
Monomorium dichroum Forel, 1902: 212. Syntype workers: Poona, India (Wroughton) (BMNH, CASENT0902222) [examined]; Bombay, India (Wroughton) (MHNG, CASENT0908718) [examined] **syn. nov.**

##### Distribution.

**Figure 6.**
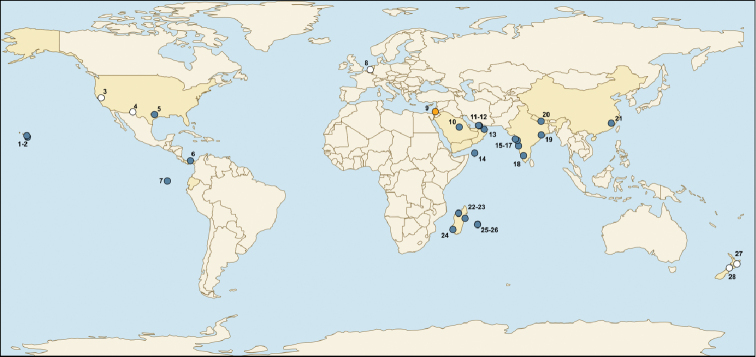
Distribution of *Monomorium sahlbergi*. White circles represent interceptions; orange circle represents type locality. Details of map locations are given in Appendix 2.

All records of *M. sahlbergi* originate from desert-like, urban, industrial, and military areas ranging from sea level to an elevation of 1800 m. It is not clear from our research what the original geographic region of *M. sahlbergi* was. Based on the distribution of other species in the *salomonis* group, the native distribution would include specimens from the Indomalaya region (Nepal, India, Thailand). Our data came from the following main geographic regions: Palearctic (China, Israel, Netherlands (interception)), Australian (New Zealand, from likely interceptions), Nearctic (USA, in part interceptions), Neotropical (Panama, Galapagos), Afrotropical (Reunion, Madagascar) and Oceania (Hawaii) (Fig. 6).

## Discussion

The global distribution of *Monomorium sahlbergi* suggests a history of introductions. Although the native distribution requires further evaluation, specimen records from disturbed habitats suggest that, like the introduction in the Netherlands, this species has already been introduced to other regions. Some distribution records suggest that *M. sahlbergi* could indeed be a successful invasive species, and is already successfully established in areas such as disturbed areas on the islands of the Galapagos (Ecuador) and urban areas in Texas, USA, Panama-City, Hawaii, Madagascar, and Reunion.

It is easy to confuse *M. sahlbergi* with the well-known pharaoh ant *M. pharaonis*, because the former also lives near or in human settlements and looks very similar to *M. pharaonis*. Therefore, we suspect that *M. sahlbergi* has more than once been misidentified as *M. pharaonis*, a view supported by the misidentifications encountered in this study. These findings suggest that *M. sahlbergi* is likely more common than we realise.

## Supplementary Material

XML Treatment for Monomorium sahlbergi

## Appendix 1

**Table:** Monomorium and outgroups COI sequences information. Asterisks (*) in the final column indicate duplicated haplotypes not included in the phylogeny.

Species	Specimen ID number	Geographical region	GenBank accession number	BOLD process ID number	
*Monomorium aithoderum*		Australia	KJ847507		
		Australia	KJ847472		
		Australia	KJ847473		
*Monomorium emarginatum*		USA	KR783702		
		USA	MG458955		
*Monomorium exiguum*	CASENT0135941-D01	Madagascar	MT887664	ASNAU286-09	
	CASENT0145058-D01	Madagascar	MT887665	ASNAU751-09	
	CASENT0147596-D01	Madagascar	MT887669	ASNAU850-09	*
*Monomorium fieldi*		Australia	JQ846291		
		Australia	JQ846292		
*Monomorium floricola*	CASENT0136664-D01	Comoros	GU709847	ASANO792-09	
		Costa Rica	KC418239	ACGAJ207-11	
*Monomorium minimum*		USA	KX687942		
		USA	KX687943		
*Monomorium pharaonis*		Africa	KJ847499		
		Costa Rica	KC419188	ACGAJ043-11	
		Korea	KC407745		
	CASENT0078602-D01	Madagascar	GU710435	ASANP639-09	
	CASENT0120402-D01	Madagascar	GU710434	ASANP651-09	*
	CASENT0120437-D01	Madagascar	GU710437	ASANP653-09	*
	CASENT0120835-D01	Madagascar	GU710436	ASANP657-09	*
	CASENT0122487-D01	Madagascar	GU710439	ASANP666-09	*
	CASENT0122499-D01	Madagascar	GU710438	ASANP667-09	*
	CASENT0123480-D01	Madagascar	GU710441	ASANP675-09	*
	CASENT0125050-D01	Madagascar	GU710440	ASANP679-09	
	CASENT0159459-D01	Seychelles	HQ546947	ASAND352-10	*
	CASENT0159472-D01	Seychelles	HQ546950	ASAND355-10	*
	CASENT0160217-D01	Seychelles	HQ546997	ASAND421-10	*
	CASENT0160424-D01	Seychelles	HQ547018	ASAND445-10	*
	CASENT0161400-D01	Seychelles	HQ547092	ASAND546-10	*
	CASENT0162033-D01	Seychelles	HQ547099	ASAND557-10	
	CASENT0162075-D01	Seychelles	HQ547102	ASAND560-10	
		USA	JX402725		
		USA	KC617835	DIRTT037-11	
*Monomorium rothsteini*		Australia	KC572958		
		Australia	KC572978		
*Monomorium sahlbergi*	RMNH.5082323	Israel	MT943758	MONOM001-20	
	CASENT0118484-D01	Madagascar	MT887671	ASAMY032-07	
	CASENT0121143-D01	Madagascar	GU709866	ASANO669-09	
	CASENT0122926-D01	Madagascar	GU709869	ASANO683-09	
	CASENT0122935-D01	Madagascar	GU709868	ASANO684-09	*
	CASENT0122940-D01	Madagascar	GU709871	ASANO685-09	*
	CASENT0122947-D01	Madagascar	GU709870	ASANO686-09	
	CASENT0122966-D01	Madagascar	GU709873	ASANO687-09	
	CASENT0122991-D01	Madagascar	GU709872	ASANO688-09	*
	CASENT0124847-D01	Madagascar	GU709875	ASANO692-09	
	CASENT0009847-D01	New Zealand	MT887667	ASAMI149-07	
	CASENT0125184-D01	Reunion	MT887663	ASAMY578-07	
*Monomorium sahlbergi*	CASENT0125194-D01	Reunion	MT887660	ASAMY580-07	*
	CASENT0125332-D01	Reunion	MT887666	ASAMY588-07	
	CASENT0125334-D01	Reunion	MT887662	ASAMY590-07	*
	CASENT0125339-D01	Reunion	MT887661	ASAMY591-07	*
	RMNH.5082322	Netherlands	MT943757	MONOM002-20	
	CASENT0191347-D01	USA (Hawaii)	MT887670	ASANE610-10	
	CASENT0191348-D01	USA (Hawaii)	MT887668	ASANE611-10	*
*Monomorium sordidum*		Australia	KC573031		
		Australia	KJ956922		
*Monomorium stictonotum*		Australia	KJ847503		
*Monomorium termitobium*	CASENT0157182-D01	Madagascar	JN283174	ASANH122-10	
*Huberia striata*		New Zealand	FJ824424		
*Podomyrma* sp.		Australia	DQ353334		

## Appendix 2

**Table:** Distribution details of the mapped specimens of *Monomorium sahlbergi*.

Map ID number	Locality	Country	Latitude	Longitude
1	Maui	USA	20.63	-156.1
2	Kawaihae	USA	20.04	-155.8
3	Elk Grove	USA	38.41	-121.3
4	El Paso	USA	31.76	-106.4
5	College Station	USA	30.63	-96.33
6	Panama City	Panama	8.98	-79.52
7	Galapagos	Ecuador	-0.59	-90.32
8	Rijen	Netherlands	51.59	4.92
9	Jericho	Israel	31.86	35.46
10	Riyadh	Saudi Arabia	24.71	46.68
11	Um-al-Quwain	UAE	25.52	55.71
12	Wadi Maidaq	UAE	25.35	56.09
13	Muscat	Oman	23.59	58.41
14	Socotra	Yemen	12.46	53.82
15	Mumbai	India	19.08	72.88
16	Pune	India	18.4	73.85
17	Belgaum	India	15.85	74.5
18	Coonoor	India	11.35	76.8
19	Odisha	India	20.95	85.1
20	Hetauda	Nepal	27.44	85
21	Fujian	China	26.48	117.92
22	Mahajanga	Madagascar	-15.69	46.33
23	Toamasina	Madagascar	-18.14	49.4
24	Toliara	Madagascar	-23.35	43.69
25	Grotte des Premiers Français	Reunion	-21.02	55.26
26	Le Port	Reunion	-20.94	55.3
27	Napier Port	New Zealand	-39.48	176.91
28	Port Nelson	New Zealand	-41.26	173.28
